# Human-centered incubator: beyond a design concept

**DOI:** 10.1038/jp.2013.117

**Published:** 2013-11-26

**Authors:** R H M Goossens, H Willemsen

**Affiliations:** 1 Faculty of Industrial Design Engineering, Delft University of Technology, Delft, The Netherlands; 2 BabyBloom Healthcare, Leiden, The Netherlands

We read with interest the paper by Ferris and Shepley^1^ on a human-centered design project with university students on neonatal incubators. It is interesting to see that in the design solutions and concepts as presented by Ferris and Shepley,^1^ human-centered design played an important role.

In 2005, a master thesis project was carried out in the Delft University of Technology, following a similar human-centered design approach.^2, 3^ In that design project we also addressed the noise level inside the incubator, as several studies^4, 5^ have found that incubators' climate systems itself cause sound levels inside the incubator far above 45 dBA, as recommended by the American Academy of Pediatrics.^6^

The resulting incubator concept, called the BabyBloom Incubator, incorporates many of the features that were presented in the concepts of the mentioned article (for example, sound monitoring and camera feed), although in the field of ergonomics more radical design decisions were made (for example, possibility for full placement over mother's bed).

Recently, the final design received CE certification, and since that point on it is available on the European market. Therefore, we think that the statement that ‘the fundamental design of the incubator has remained largely unchanged for at least 30 years' is obsolete.
